# Revolutionizing microbial treasure troves: innovative strategies for natural products discovery

**DOI:** 10.1007/s13659-025-00565-0

**Published:** 2026-01-10

**Authors:** Yu-Jie Li, Ming-Hua Qiu, Xing-Rong Peng

**Affiliations:** 1 State Key Laboratory of Phytochemistry and Natural Medicines, Kunming Institute of Botany, Chinese Academy of Sciences, Kunming, 650201 China; 2 Kunming College of Life Science, University of Chinese Academy of Sciences, Kunming, 650204 China; 3 Key Laboratory of State Forestry and Grassland Administration on Highly-Efficient Utilization of Forestry Biomass Resources in Southwest China, Southwest Forestry University, Kunming, 650224 People’s Republic of China

**Keywords:** Microbial natural products, OSMAC strategy, Genome mining, Machine learning

## Abstract

Microorganisms represent Earth's most abundant biological resource, producing metabolites of immense value across medicine, agriculture, and industry. Conventional cultivation and screening techniques, however, suffer from inefficiency and fail to meet contemporary demands. Providing a comprehensive overview, this review details how the One Strain Many Compounds (OSMAC) strategy—addressing cultivation bottlenecks—and genomics-driven mining approaches are revolutionizing the discovery of novel microbial metabolites. Crucially, it underscores the broad adoption of innovative technologies like machine learning to enable faster, more effective gene and structure targeting. Synthesizing case studies from 2019 to 2025, the review catalogs newly identified compounds and their bioactivities, while outlining future research directions to establish a theoretical framework for efficient microbial natural product exploration. These advanced discovery strategies are significantly accelerating the identification of structurally diverse lead compounds with novel mechanisms of action, thereby revitalizing pipelines for new antibiotic, anticancer, and therapeutic drug development.

**Graphical abstract:**

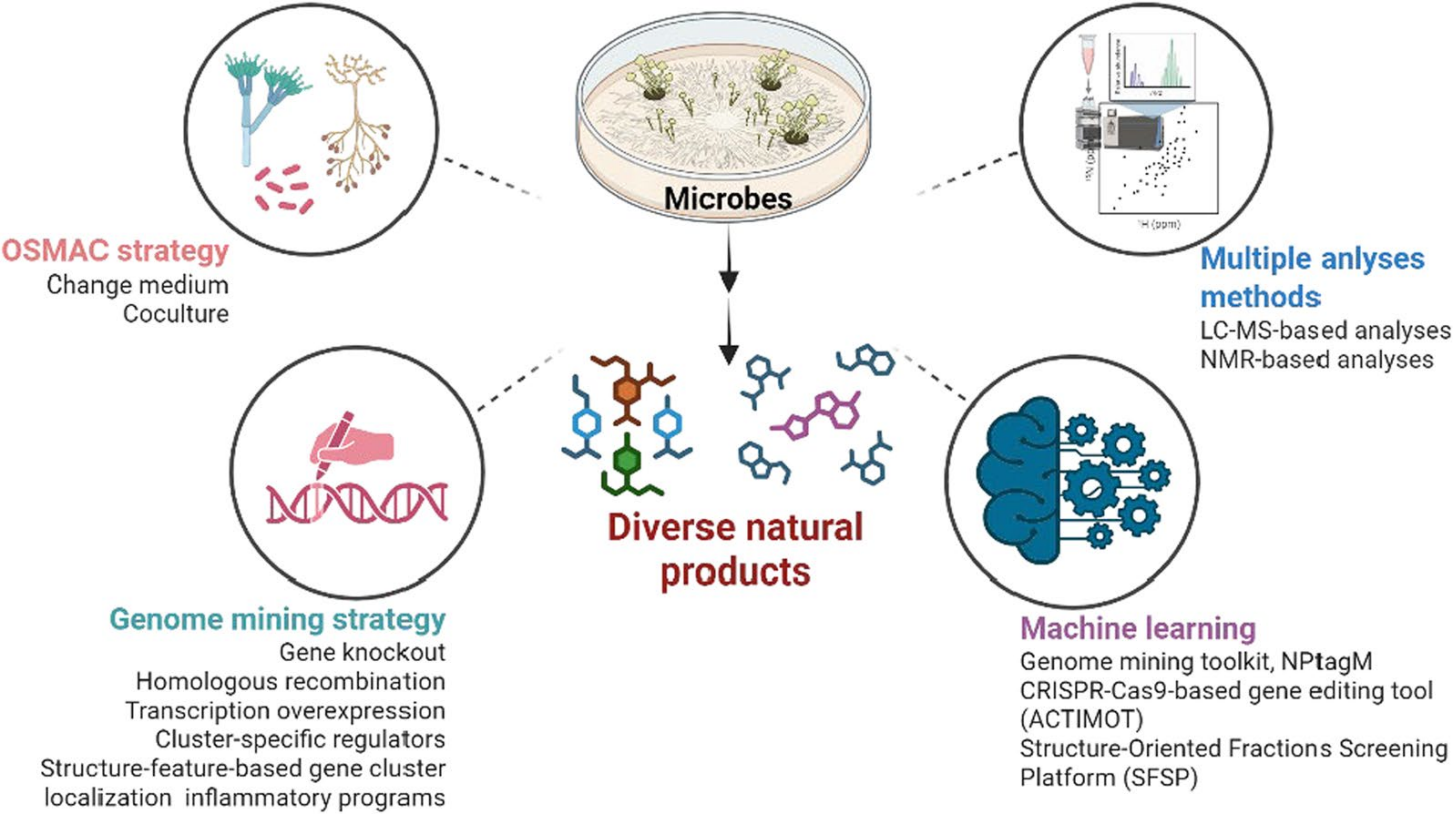

**Supplementary Information:**

The online version contains supplementary material available at 10.1007/s13659-025-00565-0.

## Introduction

**Fig. 1:**
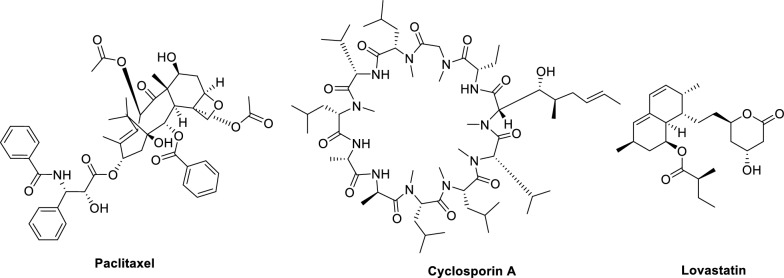
Chemical structures of significant microbial natural drugs (paclitaxel, cyclosporin A, and lovastatin)

Microbial natural products, produced by diverse microorganisms including bacteria, fungi and actinomycetes, are renowned for their unique structural characteristics and broad spectrum of biological activities. These compounds have long been recognized as a crucial source of lead compounds in pharmaceutical development [1]. Statistical data reveal that approximately 70% of clinically used antibiotics originate from microbial sources [2, 3]. Beyond their antibiotic applications, microbial metabolites have also contributed significantly to the development of various therapeutic agents, including anticancer drugs. A notable example is paclitaxel, which was initially isolated from plants but is now commercially produced through microbial fermentation [4, 5]. Furthermore, microbial-derived compounds such as the immunosuppressant cyclosporin A (essential in organ transplantation) and the cholesterol-lowering agent lovastatin [6, 7] have become indispensable in modern medicine (Fig. 1).

Despite the remarkable potential of microbial metabolites, researchers face several persistent challenges: (1) Cultivation limitations: More than 90% of environmental microorganisms resist cultivation under standard laboratory conditions, leaving an enormous reservoir of potentially novel compounds unexplored—particularly from unculturable microbes inhabiting extreme environments such as deep-sea ecosystems. (2) High rediscovery rate: Frequently studied microbial strains have been extensively characterized, resulting in inefficient screening processes that predominantly yield known compounds. (3) Silent biosynthetic gene clusters (BGCs): While microbial genomes typically harbor numerous BGCs, the majority remain transcriptionally inactive under conventional laboratory conditions, rendering their associated metabolic products undetectable. These challenges underscore the urgent need for paradigm-shifting innovations that transcend traditional research approaches, demanding the development of novel methodologies to unlock the full potential of microbial resources.

Unlike previous reviews limited to singular methods (e.g., synthetic biology or metabolomics) [8–11] or compound classes (e.g., peptides/polyketides) [12], this work provides a comprehensive analysis of challenges in discovering structurally diverse, bioactive microbial natural products and the innovative strategies addressing them. It systematically examines three transformative approaches: "One Strain Many Compounds" (OSMAC) cultivation, genome mining (CRISPR-enhanced), and machine learning-driven platforms (NPtagM/SFSP), curating advances (2019–2025) yielding over 300 novel bioactive compounds. The review concludes by proposing a roadmap integrating single-cell analysis, multi-omics, and synthetic biology to unlock microbial "dark matter" for next-generation drug/agrochemical discovery.

## Innovative strategies

### OSMAC strategy

**Fig. 2:**
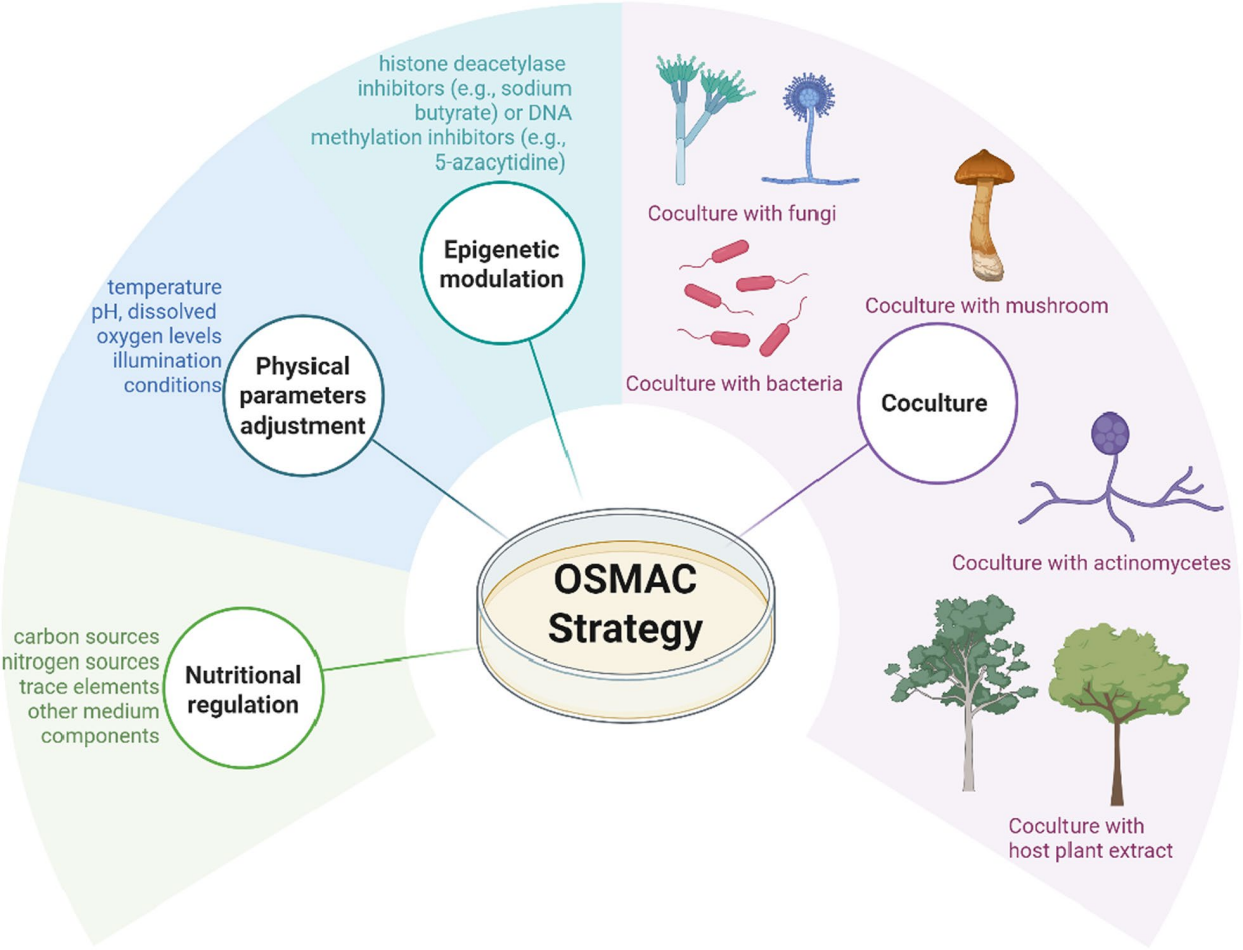
The description of OSMAC strategy

The "One Strain Many Compounds" (OSMAC) strategy represents an innovative approach in microbial cultivation optimization, which systematically modulates culture conditions to activate silent BGCs in microorganisms, thereby significantly expanding the diversity of their secondary metabolites [13]. In conventional microbial natural product research, a single strain typically yields only a limited array of known compounds under standard laboratory conditions. In contrast, the OSMAC approach substantially enhances chemical diversity through strategic manipulation of cultivation parameters. The core principles of OSMAC involve: (1) Nutritional regulation: Modifying carbon sources, nitrogen sources, trace elements, and other medium components to redirect metabolic flux; (2) Physical parameter adjustment: Optimizing temperature, pH, dissolved oxygen levels, and illumination conditions; (3) Biological interactions: Employing co-culture systems to simulate natural ecological interactions, thereby inducing the production of defensive metabolites through competitive or symbiotic relationships; (4) Epigenetic modulation: Utilizing histone deacetylase inhibitors (e.g., sodium butyrate) or DNA methylation inhibitors (e.g., 5-azacytidine) to activate dormant gene clusters. This multi-parameter synergistic regulation strategy establishes a novel paradigm for the exploration of microbial natural products (Fig. 2).

#### Modification of cultivation methods

##### Variations in culture medium

**Fig. 3:**
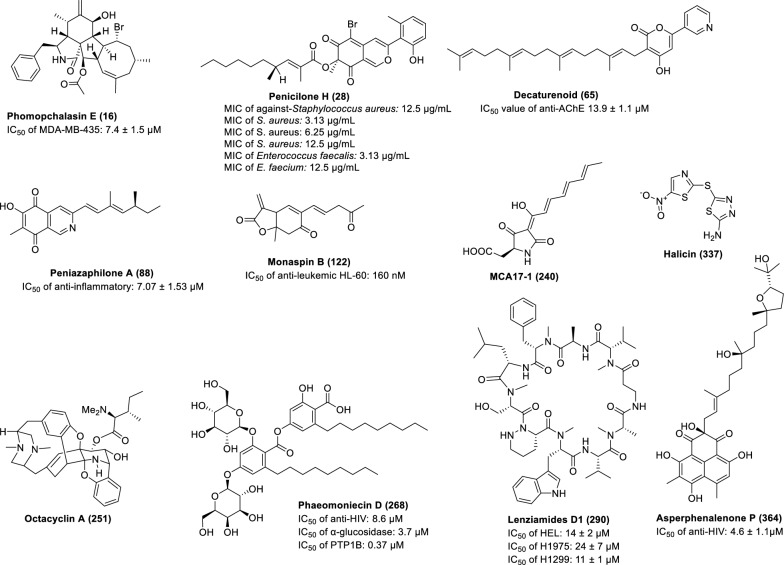
Structures of representative bioactive compounds (other compounds are shown in supplementary files) obtained from the coculture

Targeted modification of culture medium components can specifically activate fungal secondary metabolic pathways. Research demonstrates that: the marine fungus *Pseudallescheria boydii* F44-1 produces novel spirocyclic bisindole alkaloids pseudboindoles A-B (**1‒2**) in amino acid-supplemented GPY medium [14], while *Trichocladium* sp. yields the first-reported bismacrolactone 13-*N*-(2-carboxyphenyl) colletoketol (**3**) in rice medium containing 2% tryptophan [15]. In the cultivation of the endophytic fungus *Aplosporella javeedii*, the addition of 3.5% NaNO_3_ induced the production of seven new compounds, aplosporellins A-C (**4‒6**), E–G (**7‒9**) and J (**10**), while 3.5% monosodium glutamate yielded four structurally distinct compounds, aplosporellins D (**11**) /H (**12**) /I (**13**) /K (**14**), highlighting the specific regulatory effects of nitrogen sources on metabolic pathways [16]. The mangrove fungus *Phomopsis* sp. QYM-13 produced brominated cytochalasins, phomopchalasins D (**15**), E (**16**), I (**17**), K (**18**), and N (**19**) in medium containing 3% NaBr, while iodinated derivatives, phomopchalasins F (**20)**, G (**21**), H (**22**), J (**23**), L (**24**), M (**25**), O (**26**) were generated in 3% KI medium. Notably, compounds **16**, **20**, and **22** represented the first brominated and iodinated cytochalasins, exhibiting significant cytotoxicity against MDA-MB-435 cancer cells [17]. Similarly, *Penicillium janthinellum* HK1-6 produced two novel brominated azaanthraquinones penicilones G**-**H (**27‒28**) (Fig. 3 and Additional file 1: Fig. S1) and two new tricyclic polyketides penijanthinones A**-**B (**29‒30**) under NaBr induction, with penicilone H (**28**) showing antimicrobial activity against various pathogens (MIC: 3.13–12.5 μg/mL) [18]. These findings systematically reveal how medium components (amino acids, nitrogen sources, halide salts) precisely regulate the structural diversity and bioactivity of microbial metabolites, providing crucial strategies for targeted discovery of novel bioactive lead compounds.

The coral-derived fungus *Stachybotrys chartarum* produced six new phenyldrimanes, distachydrimanes A-F (**31‒36**), with unprecedented C-18-C-23 connectivity in medium containing 0.1% Fe_2_(SO_4_)_3_ and 3% sea salt. The compounds **31**, **35**, and **36** inhibited L1210 cells through multiple mechanisms: > 70% inhibition rate, G0/G1 phase arrest, senescence promotion, and mitochondrial apoptosis pathway activation [19]. Meanwhile, *Preussia isomera* yielded the rare tetrahydro-2*H*-1,2-oxazine alkaloid ( ±)**-**preisomide (**37**) when cultured in rice medium supplemented with wheat bran liquid medium. In MEB-containing rice medium, it produced the sesquiterpenoid enantiomers ( ±)-preuisolactone A (**38**), featuring a novel tricyclo [4.4.0^1,6^.0^2,8^] decane skeleton, with a MIC of 10.2 μM against *Micrococcus luteus* [20, 21]. These findings systematically demonstrate how the mixed composition of culture medium precisely modulates both the structural diversity and bioactivities of microbial metabolites, thereby establishing an innovative research paradigm for targeted discovery of bioactive molecules through optimized culture conditions.

Research on the medium type-dependent activation of fungal secondary metabolites demonstrates that: the corn-derived *Fusarium* sp. YMX-C33 produced six novel sesquiterpenoids, fusarchlamols A-F (**39‒44**), in PDB medium, while coffee medium specifically produced methyltricinonoate (**45**), a structurally novel natural sesquiterpenoid, along with compounds **41** and **42**. Bioactivity assessment demonstrated that compounds **39**, **40**, and **43**/**44** exhibited significant inhibitory activity against the coffee pathogenic fungus *Alternaria alternata* (MIC: 1 μg/mL) [22]. Research on *Penicillium canescens* found that solid rice medium produced penicanesins A**-**B (**46‒47**), and E**–**G (**48‒50**), while liquid medium specifically yielded penicanesins C**-**D (**51–52**). Remarkably, ( ±)-penicanesins A (**46**) and B (**47**) featured a rare 6/6/6/6 heterotetracyclic skeleton, with ( +)-penicanesin A (**46**) showing IC_50_ values of 8.2 and 9.8 μM against HL-60 and SW480 cancer cells, respectively [23]. Similarly, *Pleotrichocladium opacum* produced compounds **53–54** in JSA liquid medium, compound **55** in rice medium, and compound **56** in wheat medium [24]. These findings systematically demonstrate how medium type and physical state (solid/liquid) precisely regulate the structural diversity and bioactivity of microbial metabolites. This approach provides a strategic framework for the targeted discovery of novel bioactive lead compounds.

##### Chemical epigenetic modifications

Chemical epigenetics refers to the exogenous addition of epigenetic modifiers, including histone deacetylase (HDAC) inhibitors and DNA methyltransferase (DNMT) inhibitors, to specifically alter the epigenetic modification state of microbial chromatin [25]. This approach alleviates gene silencing effects, activates dormant BGCs, and promotes the biosynthesis of structurally novel bioactive compounds [26, 27]. Within the OSMAC strategy, chemical epigenetic regulation primarily functions through the following molecular mechanisms: HDAC inhibitors (such as sodium butyrate, suberoylanilide hydroxamic acid, SAHA and suberic bis-hydroxamic acid, SBHA) increase histone acetylation levels, loosening condensed chromatin structures and significantly enhancing the transcriptional activity of secondary metabolite genes [28, 29]; DNMT inhibitors (such as 5-azacytidine, 5-Aza) reduce DNA methylation levels, effectively reversing gene silencing and activating cryptic metabolic pathways [30, 31]; histone methylation modulators finely regulate gene expression by dynamically adjusting histone methylation marks (e.g., activating H3K4me3 or repressive H3K27me3 modifications) [32].

The research team successfully activated silent metabolic pathways in the endophytic fungus *Chaetomium globosporum* isolated from *Euphorbia humifusa* using 5-Aza, leading to the isolation of two novel cyclopentenones globosporins A-B (**57‒58**) and two monoterpene indole alkaloids globosporines C-D (**59‒60**). Notably, compound **60** exhibited significant inhibitory activity against *Xanthomonas oryzae* (MIC: 14‒72 μg/mL), demonstrating excellent potential for development as a biopesticide in both in vitro and in vivo tests [33].

Treatment of the endophytic fungus *Penicillium* sp. KMU18029 with SAHA yielded two pairs of diterpene hybrids, pyrandecarurins A-B (**61‒62**) and pileotins A-B (**63–64**), which feature unique pyridopyrrolidone-decaturene/oxalic acid hybrid scaffolds. Of particular interest, the potential precursor decaturenoid (**65**) showed moderate activity against AChE with an IC_50_ value of 13.9 ± 1.1 μM [34].

The combined application of SBHA and 5-Aza on the deep-sea-derived fungus *Eutypella* sp. MCCC 3A00281 resulted in the production of 17 novel sesquiterpenoids eutypeterpenes A-Q (**66–82**). Among these, compound **66** represents the first bergamotane-type sesquiterpene containing a dioxolane unit, while compounds **80–82** constitute an entirely new subclass of sesquiterpenes with cyclopentane structures. All compounds showed weak cytotoxic activity with IC_50_ > 100 μM on RAW 264.7 cells, indicating safety to macrophages [35]. Similarly, the combined treatment of marine-derived fungus *Aspergillus versicolor* XS-20090066 with SAHA and 5-Aza, leading to enhanced production of diverse secondary metabolites. Through an integrated approach combining GNPS with bioactivity-guided fractionation, three previously undescribed compounds were characterized: the nucleoside derivatives kipukasins K (**83**) and L (**84**), along with the sesquiterpenoid aspergillusene E (**85**). Antimicrobial evaluation revealed remarkable potency, with compounds **83** and **85** demonstrated significant antibacterial activity against *Staphylococcus aureus* and other pathogens (MIC: 8‒16 μg/mL), providing promising candidates for anti-infective drug development [36].

The collective findings demonstrate that precisely designed epigenetic strategies can effectively activate silent biosynthetic pathways in microorganisms across diverse ecological niches, yielding structurally novel secondary metabolites with significant biological relevance.

#### Coculture

##### Fungal-fungal co-culture

Recent advances in fungal co-culture technology have revolutionized natural product discovery by effectively mimicking microbial ecological interactions [37–39]. This approach activates silent BGCs in fungi, enabling the production of structurally novel secondary metabolites that are typically undetectable in monoculture systems. Notably, the method not only expands chemical diversity but also enhances yields of target compounds, thereby generating valuable resources for pharmaceutical lead development. As a cutting-edge strategy in microbial natural product research, this technology demonstrates considerable potential for drug discovery applications.

1. Co-culture with *Penicillium* fungi

*Penicillium* species have been extensively studied as prolific producers of structurally diverse and biologically active natural products [40, 41]. Recent genomic analyses reveal these filamentous fungi possess numerous silent biosynthetic gene clusters encoding potential secondary metabolites [42]. Through culture optimization and epigenetic modulation strategies, researchers have successfully activated these cryptic pathways to discover novel antibiotics, antitumor agents, and other pharmacologically relevant compounds from *Penicillium* strains [43].

Co-culture of the marine-derived *P. bilaiae* MA-267 and *P. chermesinum* EN-480 produced two new terpenoid derivatives, chermebilaenes A-B (**86–87**). Compound **86**, as the first hybrid of acorane-type sesquiterpene and octadecadienoic acid, exhibited strong inhibitory activity against *Ceratobasidium cornigerum* and *Edwardsiella tarda*[44]. Meanwhile, the co-culture system of mangrove endophytic fungi *P. sclerotiorum* THSH-4 and ZJHJJ-18 produced nine new azaphilone derivatives, peniazaphilones A-I (**88–97**). Compound **88**, the first naturally occurring isoquinolinequinone-structured azaphilone, demonstrated superior anti-inflammatory activity compared to indomethacin and showed significant cytotoxicity against A549 and MDA-MB-435 tumor cells [45] (Fig. 3 and Additional file 1: Fig. S1). Additionally, co-culture of the extremophilic *P. fuscum* with *P. camembertii/clavigerum* activated the production of berkeleypenostatins A-G (**98–103**), among which compound **101** exhibited broad-spectrum antitumor activity (TGI 1–10 μM) and the ability to inhibit the migration of human pancreatic cancer cells [46]. Another study involving *P. bialowiezense* and *Pestalotiopsis* sp. co-culture yielded six new prenylated chromane derivatives, including two pairs of enantiomeric ones (**104a**/**104b-105a**/**105b**) and two optical pure ones (**106–107**), as well as two new isoprenylated phenol glucoside derivatives (**108–109**), along with previously reported enantiomers **104a**/**104b** and **105a**/**105b**, with **104a**/**104b** showing significant GUS inhibitory activity [47]. Furthermore, co-culture of *P. brasilianum* MST-FP1927 and *Aspergillus nomius* MST-FP2004 led to the discovery of miktospiromide A (**110**) and kitrinomycin A (**111**), the latter of which exhibited notable inhibitory effects against mouse melanoma cells and bovine parasites [48].

(2) Co-culture with *Aspergillus* fungi

The *Aspergillus* genus encompasses a diverse and ubiquitous group of fungi renowned as prolific producers of secondary metabolites [42]. They are particularly adept at generating structurally diverse natural products with significant bioactivity [49], including renowned antibiotics, cholesterol-lowering drugs, mycotoxins, and various industrial enzymes [50–52]. Their rich repertoire of BGCs renders them a valuable treasure trove for drug discovery and biotechnological applications.

The co-culture of *A. fischeri* and *Xylaria flabelliformis* produced wheldone (**112**), which exhibited notable cytotoxicity against breast, ovarian, and melanoma cancer cells [53]. The interaction between *A. oryzae* and *Epicoccum dendrobii* not only increased kojic acid production to 1.10 g/L, but also activated the production of four novel metabolites, epiclactones A-B (**113–114**), epioxochromane (**115**) and aoergostane (**116**), with multi-omics analysis revealing that this process is closely linked to fungal defense mechanisms [54]. In the co-culture system of marine-derived *A. insulicola* IMB18-072 and *Alternaria angustiovoidea* IMB20-805, the cyclic tetrapeptides violaceotides B-E (**117–120**) were isolated and identified. Among these, compounds **118–119** demonstrated selective inhibitory activity against aquatic pathogens (*Edwardsiella tarda* and *E. ictaluri*), while **117–120** significantly suppressed the expression of the inflammatory factor IL-6 [55]. The co-culture system of *A. oryzae* and *Monascus purpureus* successfully yielded two novel cyclohexyl-furan compounds, monaspins A-B (**121–122**). Compound **122** demonstrated remarkable anti-leukemic HL-60 cell activity (IC_50_: 160 nM) and exhibited significant in vivo anti-leukemic effects in a mouse leukemia model by reducing white blood cell, lymphocyte, and neutrophil counts [56] (Fig. 3 and Additional file 1: Fig. S1). Additionally, the co-culture study of *Amphichorda* sp. KMM 4639 and *A. carneus* KMM 4638 yielded important results, leading to the discovery of five new quinazolinone alkaloids, felicarnezolines A-E (**123–127**), and a novel highly oxygenated chromene derivative, oxirapentyn M (**128**). Notably, compound **124** exhibited potent cardioprotective and neuroprotective effects, effectively mitigating CoCl_2_-induced damage in H9c2 cardiomyocytes and SH-SY5Y neuroblastoma cells [57]. On the other hand, the marine-derived co-culture system of *A. aculeatinus* WHUF0198 and *Penicillium* sp. DM27 achieved a major breakthrough: not only were aculeaquamides B-C (**129–130**) obtained, featuring a rare 7/6/5/5/6/5 hexacyclic scaffold, but 17 structurally novel pyranopyridone alkaloids, aculeapyridones A-Q (**131–147**), were also isolated. Among these, compounds **142–145** were particularly distinctive due to their unique *N*-methoxy group, while compounds **131–147**, **139–140**, and **142–145** demonstrated significant protective effects against acetaminophen-induced liver injury [58, 59].

(3) Co-culture of macrofungal

Macrofungi hold significant value in the fields of food, medicine, and biotechnology. In terms of food applications, they are rich in high-quality protein, dietary fiber, and various bioactive compounds (e.g., polysaccharides and triterpenoids), making them nutritious functional food ingredients [60, 61]. In pharmaceutical applications, the secondary metabolites of macrofungi exhibit remarkable pharmacological activities. For example, *Ganoderma* polysaccharides have immunomodulatory effects [62, 63], and cordycepin shows antitumor activity [64]. These properties have led to their widespread use in both traditional medicine and modern drug development. Moreover, biotechnological approaches, such as co-culture techniques, can significantly enhance the production of bioactive compounds, further expanding their application potential [65].

Co-cultivation of *Pleurotus ostreatus* SY10 and *P. eryngii* SY302 yielded the monoterpene pleurotusin A (**148**) and cyclopentenone pleurotusin B (**149**), with **148** exhibiting antibacterial activity against *Staphylococcus aureus* [66]. In another study, the co-culture of *Pleurotus ostreatus* SY10 and *Trametes robiniophila* SY636 yielded new terpenoids postredienes D-H (**150–154**), among which compound **150** exhibited inhibitory activity against *Candida albicans* and *Cryptococcus neoformans* comparable to amphotericin B [67]. When *Trametes versicolor* SY630 was co-cultured with *Vanderbylia robiniophila* SY341 or *Ganoderma gibbosum* SY1001, antimicrobial compounds tramevandins A-C (**155‒157**) and sphingolipids 17-ene-1-deoxyPS (**158**) and 1-deoxyPS (**159**), which downregulate ergosterol synthesis in *C. albicans*, were discovered [68].

(4) Co-culture of other fungal species

In the co-culture system of *Clonostachys rosea* B5-2 and *Nectria pseudotrichia* B69-1, three terpenoid derivatives—furanocochlioquinol (**160**), furanocochlioquinone (**161**), and nectrianolin D (**162**)—were isolated and characterized. In vitro antitumor activity evaluation demonstrated their significant cytotoxic effects against human leukemia HL60 cells, with IC_50_ values ranging from 0.47 to 10.16 μM [69].

The investigation of immunomodulatory compounds showed that the co-culture of *Phellinus orientoasiaticus* and *Xylodon flaviporus* yielded three structurally novel sesquiterpenoids: 6-nor-4-hydroxy-1,3(4)-distepuren-14-oic acid (**163**), 6-hydroxysterpuric acid (**164**), and 13-hydroxy-7-deoxypaneolilludinic acid (**165**). Notably, compound **163** exhibited significant enhancement of NO production in LPS-induced RAW264.7 macrophage models, suggesting its potential immunomodulatory activity [70].

In the study of antifungal activity against plant pathogens, the co-culture system of *Nigrospora oryzae* and *Irpex lacteus* produced a variety of bioactive metabolites, including two squalene-type compounds, irpenigirins A-B (**166–167**); a novel azaphilone derivative, isonigirpexin C (**168**); and two sesquiterpenoids, 5-demethyl conocenol C (**169**) and nigrosirpexin A (**170**). All of these compounds displayed potent antifungal activity against phytopathogens [71]. In addition, a structurally unique isoquinoline alkaloid, irpexine (**171**), was also isolated from the co-culture of *Irpex lacteus* and *Phaeosphaeria oryzae* [72].

The co-culture of *Colletotrichum pseudomajus* and *Daldinia eschscholtzii* yielded 16 structurally new polyketide metabolites, assigning as coldaldols A-C (**172–174**), collediol (**175**), and daldinrins A-L (**176–187**). Among these, compounds **177** and **178** featured a rare benzopyran-C7 polyketide hybrid skeleton, while compounds **179–182** exhibited novel benzopyran dimer structures. Bioactivity assessment revealed that compound **174** had significantly stronger antifungal activity than the positive control drug nystatin [73].

Furthermore, phenolic compounds phexandiols A-B (**188–189**) and ester derivatives phomesters A-C (**190–192**)  were produced by the co-culture of *Phoma* sp. YUD17001 and *Armillaria* sp. [74]. Additionally, the marine-derived fungus *Cosmospora* sp. and plant pathogen *Magnaporthe oryzae* co-culture successfully induced two new derivatives, soudanones H-I (**193–194**) [75].

 The co-culture of *Herpotrichia* sp. SF09 and *Trametes versicolor* SF09A generated 12 novel metabolites, hertramycines A-L (**195–206**). Compounds **195** and **196** were sesquiterpene-saccharide conjugates containing a xylopyranosyl group, ( ±)-**197** represented the first naturally occurring linear sesquiterpene racemate, while **201** was an α-pyrone derivative bearing a xylopyranosyl unit. In the MPP⁺-induced oxidative damage PC12 cells model, all compounds displayed dose-dependent neuroprotective effects, with compound **199** showing the most potent protection at 5 μM concentration. Mechanistic studies uncovered that compound **199** likely exereted its neuroprotective effects through modulation of the PI3K-Akt/MAPK signaling pathway, making it a potential candidate for developing novel Parkinson's disease therapeutics [76].

##### Fungal-bacterial co-culture

Currently, multiple studies have shown that the co-culture of fungi and bacteria can significantly induce the production of cryptic metabolites, which are often undetectable under monoculture conditions.

1. Co-culture of fungi and *Bacillus subtilis*

*Bacillus subtilis*, a ubiquitous Gram-positive soil and rhizosphere bacterium, is a model organism prized for its prolific secondary metabolism. It efficiently produces diverse bioactive natural products, including key antibiotics (e.g., surfactin, iturin), antimicrobial peptides, antifungal polyketides, and industrial enzymes [77]. Its remarkably modular nonribosomal peptide synthetase (NRPS) and polyketide synthase (PKS) gene clusters make it an ideal platform for both natural product discovery and synthetic biology applications [78].

In 2023, researchers isolated a new terpenoid derivative, andrastin I, from the marine fungus *Penicillium ochrochloron*. A subsequent co-culture with *B. subtilis* led to the discovery of a new butyrolactone homologue, ochrochloronic acid (**207**) [79]. Similarly, when the sponge-associated fungus *Aspergillus versicolor* was co-cultured with *B. subtilis*, a new cyclic pentapeptide, cotteslosin C (**208**), an aflaquinolone-type compound, 22-epi-aflaquinolone B (**209**), and two new anthraquinones, isoversicolorin B (**210**) and 6,8-*O*-dimethylbipolarin (**211**) were produced [80]. Furthermore, co-culture of the endophytic fungus *Trichocladium* sp. from *Houttuynia cordata* roots with *B. subtilis* induced the production of a new compound, 5-epi-pestafolide A (**212**) [15].

(2) Co-culture of fungi with other *Bacteria*

Beyond *B. subtilis*, co-culturing fungi with other microorganisms can also activate novel metabolic pathways. For example, when the wheat root-associated bacterium *Pantoea agglomerans* was co-cultured with the date palm leaf-derived fungus *Penicillium citrinum*, it produced new pulicatin derivatives, pulicatins H-I (**213‒214**), which exhibited strong antifungal activity, explaining the observed fungal growth inhibition in co-culture [81].

Additionally, co-culture of the actinobacterium *Streptomyces* sp. 13F051 with the fungus *Leohumicola minima* 15S071 yielded a novel polyketide-peptide hybrid natural product, ulleungdolin (**215**). This compound features a complex structure with rare glycosylation modifications and showed significant anti-migratory effects against MDA-MB-231 breast cancer cells [82].

##### Bacterial-bacterial co-culture

When *Streptomyces* sp. GA02 isolated from mountain soil was cocultured with *Pandoraea* sp. GA02N, it could efficiently synthesize the aromatic metabolite, gwanakoside B (**216**) containing a 6-deoxy-*α*-L-talopyranose structure. Particularly noteworthy is that the yield of this compound under coculture conditions reached 100 times that of axenic culture. This remarkable enhancement highlights the advantages of microbial coculture in secondary metabolite production [83].

By co-culturing *Streptomyces hygroscopicus* HOK021 and *Tsukamurella pulmonis* TP-B0596, a structurally novel bifunctional antimicrobial conjugate, harundomycin A (**217**) were identified. This conjugate was formed through a thioester bond between the 2,4-dihydroxy-3-aminobenzoic acid moiety of platensimycin and *N, N′*-bis(2,3-dihydroxybenzoyl)-*O*-seryl-cysteine (bisDHBA-Ser-Cys). Genomic analysis revealed that strain HOK021 possesses biosynthetic gene clusters for both platensimycin and enterobactin, while *T. pulmonis* TP-B0596 acts as an inducer to activate these metabolic pathways. Compound **217** not only retained platensimycin-like antibacterial activity against drug-resistant pathogens such as MRSA and VRE but also exhibited enterobactin-like iron-chelating capacity [84].

Two Red Sea-derived actinomycetes, *Actinokineospora spheciospongiae* EG49 and *Rhodococcus* sp. UR59 were cocultured, leading to the isolation of several angucycline-class compounds [actinosporins E (**218**), H (**219**), G (**220**), tetragulol (**221**)] and the anthraquinone compound capillasterquinone B (**222**), which were undetectable in monocultures. These compounds displayed high affinity for *Plasmodium* lysyl-tRNA synthetase (PfKRS1) and exhibited significant in vitro antimalarial activity [85].

In summary, co-cultivation strategy, by mimicking natural microbial interactions, has proven to be an effective approach for activating silent metabolic gene clusters and discovering novel natural products. Despite its remarkable success, the underlying molecular mechanisms remain poorly understood. Consequently, future research paradigms should shift toward integrating multi-omics and real-time analysis technologies to systematically elucidate interaction networks, ultimately enabling a paradigm shift from empirical methods to predictable and rational design.

#### Emerging cultivation technologies and tools

While the OSMAC strategy has successfully stimulated microbial metabolic potential by altering culture conditions, its limitation lies in its continued reliance on laboratory pure culture techniques, making it difficult to access the vast majority of unculturable microorganisms in nature. This constitutes a more fundamental challenge in the field of natural product discovery. To overcome this limitation, a series of novel cultivation methods and tools designed to simulate natural habitats have emerged, significantly expanding the diversity of cultivable microorganisms.

In situ cultivation is a technological strategy that overcomes the limitations of traditional laboratory pure culture. Its core principle involves placing microorganisms in their native natural environment for cultivation, thereby directly utilizing various ecological factors from the habitat to induce microbial growth [86]. The technique operates by using physical barriers such as semi-permeable membranes to allow free diffusion of essential chemical factors like nutrients and signaling molecules from the environment, while confining the target microorganisms within specific spaces, thus creating a controlled yet nearly natural growth microenvironment.

To achieve higher throughput and standardized cultivation, novel cultivation devices such as the iChip have been developed. The iChip is a high-throughput in situ cultivation device whose basic operational workflow involves: serially diluting environmental samples and mixing them with molten agarose, then injecting the mixture into a chip containing multiple microwells so that each microwell theoretically contains only a single microbial cell; subsequently sealing the chip with semi-permeable membranes and returning it to the source environment for cultivation [87]. The semi-permeable membranes allow free diffusion of growth factors and signaling molecules from the environment, providing the encapsulated microbial cells with a nearly natural chemical environment while physically isolating them within the microwells to form monoclonal colonies. Research has shown that the microbial isolation rate achieved using the iChip can be several times to an order of magnitude higher than traditional plate culture methods, thereby successfully unlocking a vast reservoir of previously "uncultured" microbial taxa and leading to the discovery of active molecules with novel structures, including antibiotics [87, 88].

Beyond the iChip, other microenvironment control technologies such as microfluidic cultivation chips [89], and microbial encapsulation [90] are rapidly developing. These emerging technologies, combined with OSMAC strategies and genome mining, together form a powerful toolkit that is systematically addressing the challenge of microbial uncultivability, paving a broad pathway for the discovery of novel natural products from microbial "dark matter."

### Genome mining strategy

**Fig. 4:**
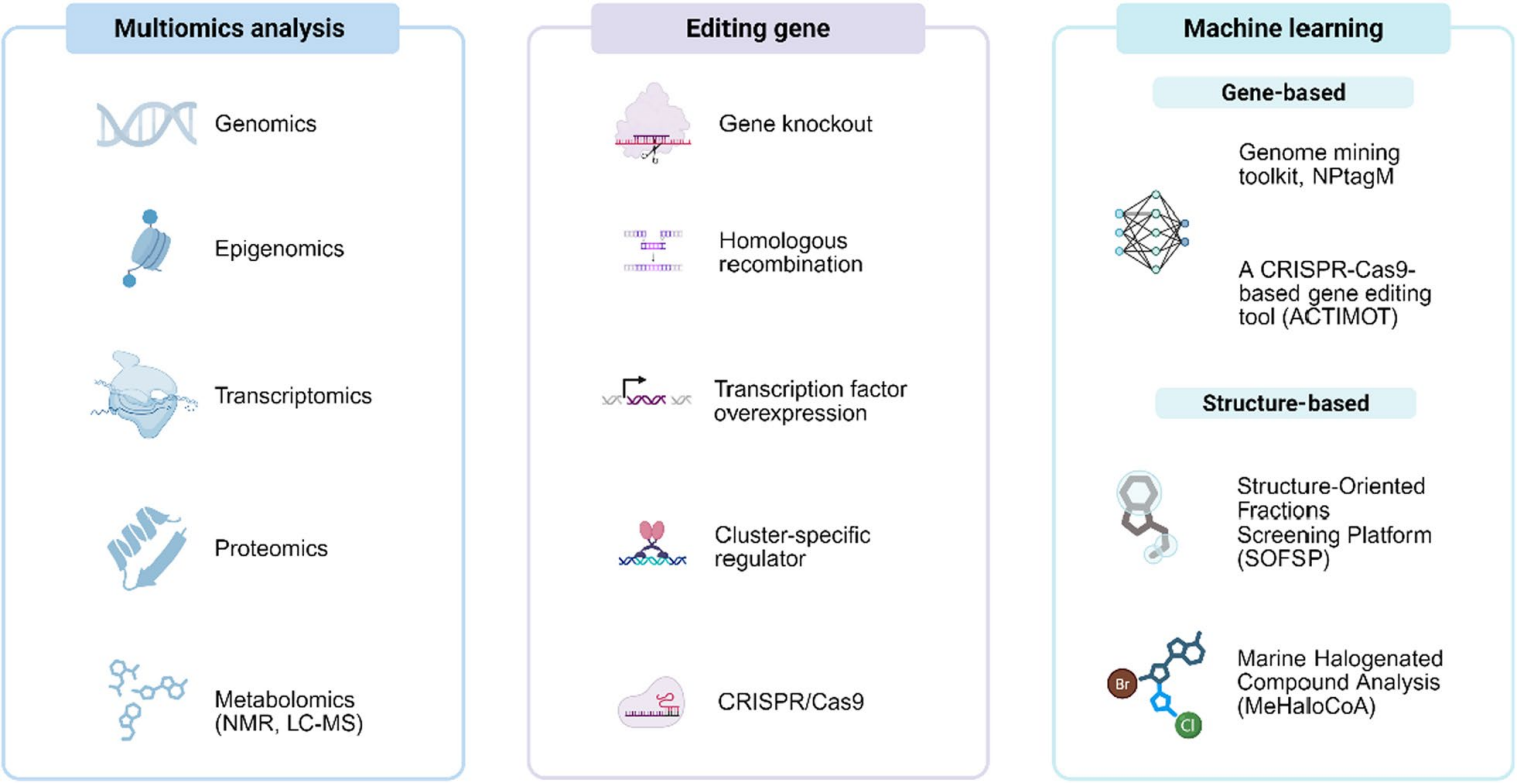
Fundamental methods of genome mining strategy

Genome mining is an integrated research strategy for natural product discovery based on microbial genomic data. The genome mining strategy is not a single research method, but rather a combinatorial research approach that continues to evolve. Depending on the specific circumstances of different research subjects, genome mining employs various methods. In 2009, Christophe Corre systematically elaborated on the genome mining strategy in a review published in *Natural Product Reports* [91]. It is notably that the rapid development of gene-editing technologies such as CRISPR [92] and Multiplex Base-Editing [93], combined with metabolic engineering modifications, has significantly enhanced both the discovery and production capacity of microbial secondary metabolites, providing robust technical support for natural product development (Fig. 4).

#### Gene knockout

Precise elimination of specific gene functions through frame-shift mutations or premature stop codons, serving as foundational tools for metabolic flux redirection. In the polar strain *Eutytella* sp. D-1, researchers employed gene knockout technology to delete the key gene responsible for triterpene cyclase biosynthesis, inducing metabolic flux reprogramming and activating dormant secondary metabolic gene clusters. In this study, 8 novel structural compounds, **223–230** were isolated and identified, among which compound **229** contained a unique 5/10 macrocyclic ether skeleton. In-depth functional studies demonstrated that the acorane-type sesquiterpenoid **230** exhibited significant anti-inflammatory activity in both cellular and transgenic zebrafish models by regulating the MAPK and NLRP3/caspase-1 signaling pathways [94].

Meanwhile, a novel antifungal compound alligamycin A (**231**) was obtained from the rapamycin-producing strain *Streptomyces iranensis* through genome mining. Targeted gene editing was used to knockout the *aliA* and *aliH* genes within the polyketide synthase (PKS) gene cluster, confirming the biosynthesis origin of the cluster. Compound **231** features a unique *β*-lactone-[6, 6]-spiroketal hybrid scaffold (with 13 chiral centers) and a novel polyketide extender unit (7-oxo-octylmalonyl-CoA). The gene knockout directly abolished the biosynthesis of the target compound. Subsequent structural derivatization (disruption of the *β*-lactone ring in alligamycin B (**232**)) demonstrated that this moiety is the essential pharmacophore for inhibiting drug-resistant fungi (including *Aspergillus* and *Talaromyces* species). Proteomics analysis revealed that compound **231** exerted its antifungal effects by compromising fungal cell wall integrity, providing a promising candidate to address the clinical limitations of current antifungal therapeutics [95].

#### Homologous recombination

Template-directed DNA repair mechanism achieving scarless gene replacement or insertion, critical for stable pathway refactoring. In the fungal strain *Calcarisporium arbuscula* NRRL 3705, researchers pioneered a novel approach by employing homologous recombination to generate the *ΔaurA* knockout mutant of the aurovertins biosynthetic gene cluster. Through high-throughput screening, two highly efficient promoters, aurBp and A07068p were identified. This integrated strategy accomplished dual objectives: it successfully activated the expression of endogenous polyketide synthase (PKS) gene clusters while simultaneously enabling high-efficiency heterologous expression of three exogenous fungal PKS gene clusters. The methodology substantially broadened the structural diversity of aurovertin-class polyketide **233**, thereby establishing an innovative technological platform for natural product structural engineering [96].

#### Transcription factor overexpression

Amplification of rate-limiting transcriptional actuators to overcome metabolic bottlenecks, particularly effective for silent cluster awakening. By employing whole-genome scanning and bioinformatic prediction techniques, researchers identified a gene cluster encoding a distinctive tandem polyketide synthase in *Ascomycete* sp. F53. The isolated azaphilone compound, lijiquinone (**234**), featured a unique cyclohexenone-bicyclic hybrid scaffold and showed remarkable cytotoxicity along with broad-spectrum antifungal activity [97]. Notably, in *Talaromyces* sp., overexpression of the transcription factor LutB successfully activated a cryptic metabolic pathway, yielding five structurally novel sclerotiorin-type azaphilones (**235–239**). Subsequent gene knockout and isotope labeling experiments elucidated the pivotal role of the LutC-LutD oxidoreductase system in the biosynthetic pathway [98].

A genetic engineering strategy was used to activate silent biosynthetic gene clusters in filamentous fungi: Using an aurovertin-deficient mutant strain as the host, overexpression of a pathway-specific zinc-finger transcription factor successfully activated the silent gene cluster *mca*17 in *Calcarisporium arbuscula*, leading to the isolation of a novel tetramic acid-type MCA17-1 (**240**). Biosynthesis of this compound was catalyzed by a PKS-NRPS hybrid enzyme, and it exhibited significant pharmacological activity in a liver fibrosis model: In the TGF-*β*-induced LX-2 hepatic stellate cell activation assay, compound **240** exhibited significant inhibitory activity to the clinical positive control drug obeticholic acid (OCA) [99] (Fig. 3 and Additional file 1: Fig. S1).

#### Engineered activation of cluster-specific regulators

Artificial triggering of pathway-specific transcriptional switches via synthetic promoters or inducer molecules, exemplified by: through comprehensive genomic mining of *Aspergillus terreus*, researchers identified a cryptic PKS-NRPS hybrid gene cluster. Activation of cluster-specific regulatory factors enabled the isolation of ten structurally novel pyranterreone, pyranterrones A-H (**241–248**), cordylactam (**249**), and 7-hydroxycordylactam (**250**). By integrating gene knockout experiments, bioinformatic analysis, and stable isotope labeling studies, the unique biosynthetic mechanism of thePKS-NRPS hybrid system was veiled in the fungal species [100].

#### Structure-feature-based gene cluster localization strategies

A fragment-guided strategy, focusing on strained cyclophane as a key biosynthetic fragment, was employed to develop a novel approach for natural product discovery. Cyclophane-containing natural products (CNPs), as a unique class of molecules widely distributed in nature, are formed through the cyclization of precursors such as polyketides or peptide chains. Their distinctive structural features confer important biological activities, like vancomycin. Of particular interest is the biosynthetic pathway of the antiviral compound herquline A [101], in which three key enzymes—HqlA (NRPS), HqlB (SDR), and HqlC (P450)—work synergistically to convert tyrosine into a dityrosine piperazine precursor and ultimately construct the strained cyclophane scaffold. Leveraging the high reactivity and structural plasticity of this cyclophane module, the researchers proposed that the HqlABC gene cluster constitutes a "minimal cyclophane-forming cassette," serving as an ideal target for discovering structurally innovative natural products. Using this method, octacycline A (**251**) was isolated from conserved gene clusters. This compound contains a complex octacyclic structure and an unprecedented hetero-[3.3.1] bicyclic framework, providing a new paradigm for expanding the structural diversity of natural products [102] (Fig. 3 and Additional file 1: Fig. S1).

#### Conservativity mining of core biosynthetic enzymes

Four important agricultural pathogenic fungi: wheat pathogens (*Bipolaris sorokiniana* and *Zymoseptoria tritici*), maize leaf spot pathogen (*Cercospora zeae*-*maydis*), and tomato leaf mold pathogen (*Fulvia fulva*) were selected for the subjects. Based on the biosynthetic characteristics of toxins such as fumonisins, the researchers targeted BGCs anchored by highly reducing polyketide synthases (HRPKSs) and identified a highly conserved BGC among these pathogens and *Trichoderma* species (including the biocontrol strain *T. afroharzianum* t-22). This BGC encoded five core enzymes: HRPKS, ABH, P450, α-glucosidase, and a predicted terpene cyclase with EH-like features. The tre BGC from *T. afroharzianum* t-22 additionally contained P450 and *O*-methyltransferase (*O*-MeT) genes. By analyzing the unique combination of these enzymes, the researchers predicted that the metabolic products might possess novel structures. The corresponding metabolite, treconorin (**252**), was successfully identified. Structural analysis revealed that this compound features a terpene-like *trans*-fused 5,7-bicyclic core skeleton, which the researchers speculate may originate from a (4 + 3) cycloaddition reaction mechanism. This discovery not only expands the structural diversity of fungal-derived natural products but also provides new insights to subsequent biosynthetic mechanism studies [103].

A "biosynthetic enzyme-guided genome mining strategy" was innovatively applied to systematically analyze conserved key biosynthetic enzymes (NRPS and IDO) of marine fungus *Neosartorya pseudofischeri* F27-1 genomes, leading to the discovery of the novel 1-benzazepine-structured alkaloid-pseudofisnins A and B (**253** and **254**) [104]. The development of this strategy stemmed from an in-depth understanding of the L-kynurenine (Kyn) metabolic pathway: although Kyn, as a key intermediate in tryptophan metabolism, is widely involved in various physiological processes, its reported use as a structural unit in natural products is extremely rare. Preliminary research revealed that all known Kyn-derived natural products (e.g., nanangelenin A and aspcandine) [105, 106] share a critical feature—their biosynthetic gene clusters invariably contain genes encoding NRPS and IDO. Building on this pattern, the research team used NRPS and IDO as molecular markers to systematically mine fungal genome databases. This approach not only successfully identified the biosynthetic gene cluster for pseudofisnins but also fully elucidated its synthetic pathway through in vivo and in vitro experiments, while characterizing the novel catalytic function of the iterative methyltransferase PseC. The successful implementation of this strategy underscores the significant value of characteristic biosynthetic enzymes as "signposts" for genome mining, providing a paradigm for the targeted discovery of rare scaffold natural products. With the continuous accumulation of fungal genome data, this mining method based on conserved features of key enzymes holds promise as a powerful tool for discovering more Kyn-derived and other types of novel bioactive molecules.

### Integration of genome mining and metabolism analysis

**Fig. 5:**
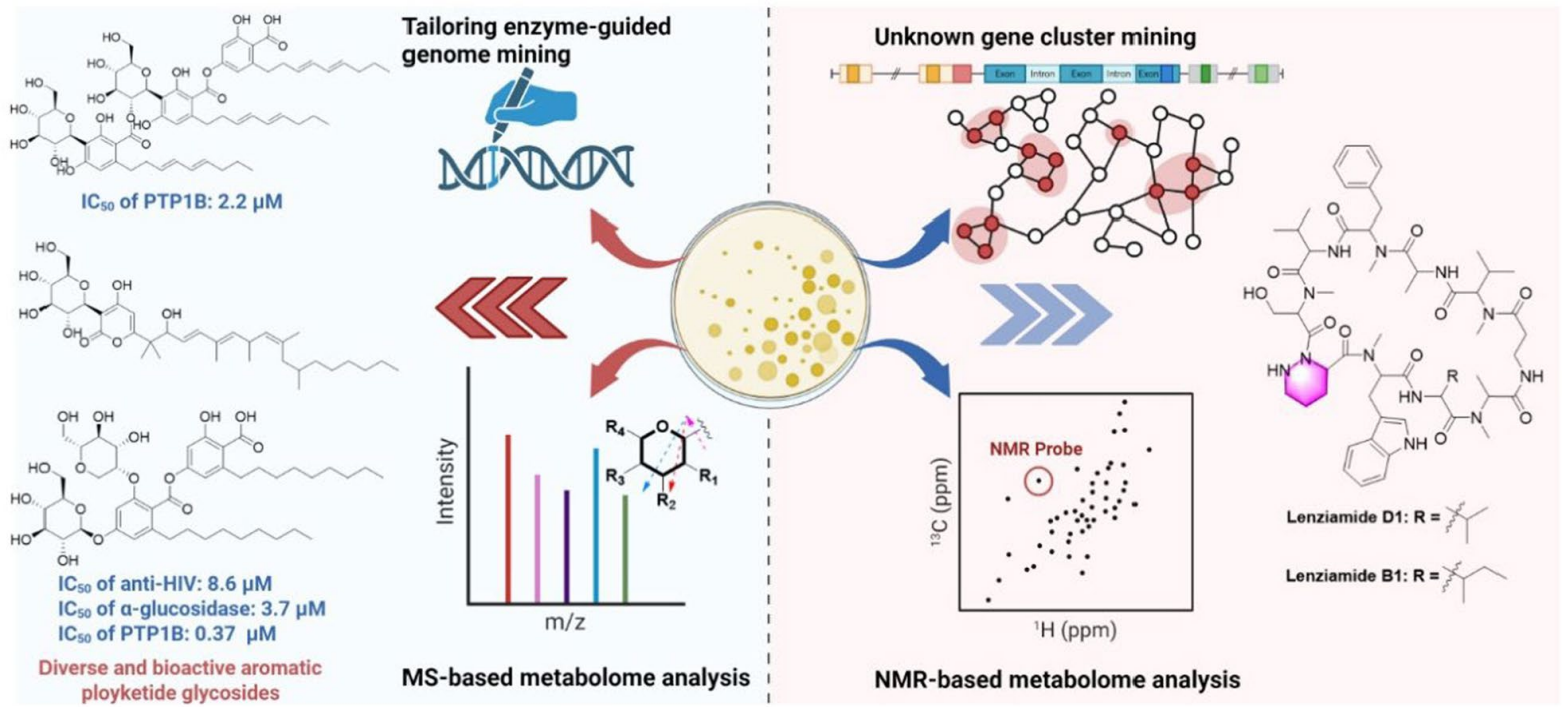
Overview of genome mining and metabolism analysis strategies

To bridge the gap between genomic predictions and tangible chemical entities, natural products uncovered through genome mining must be synergistically integrated with advanced structural analysis platforms-such as tandem mass spectrometry (LC–MS/MS), and high-resolution NMR [107–109]. This multidisciplinary convergence not only accelerates the dereplication of known compounds but also enables rapid structural elucidation of novel scaffolds, significantly compressing the discovery timeline from months to weeks. Critically, by minimizing resource-intensive isolation of redundant metabolites, this approach optimizes the path to high-value target molecules, thereby enhancing the cost-efficiency of natural product pipelines in drug development [110] (Fig. 5).

#### Integrating homology-based genome mining with HPLC–MS validation techniques

The research team developed an enzyme-guided genome mining strategy based on the key biosynthetic enzyme C-glycosyltransferase (AuCGT) involved in the antibiotic stromemycin pathway. By integrating homology-based genome mining with HPLC–MS validation techniques, they identified multiple BGCs across diverse fungal strains, leading to the characterization of 20 novel aromatic polyketide C/O-glycosides, talarocellmycins A-I (**255‒263**), carnemycin I (**264**), phaeomoniecins A-H (**265–272**) and verapyrones A-B (**273–274**). Functional characterization of three novel α-pyrone C-glycosyltransferases and their heterologous expression in *Aspergillus nidulans* enabled combinatorial biosynthesis of unnatural glycosides. Notably, these glycosides demonstrated significant antiviral, antibacterial, and antidiabetic activities [111].

Two polyketide synthase gene clusters (syw1/syw2) were identified from the marine fungus *Aspergillus sydowii* PKU374 through antiSMASH analysis. The key gene syw1G showed high homology with the known xanthone biosynthesis gene mdpG, indicating the strain's potential for xanthone production. Furthermore, researchers employed UPLC-HRESIMS/MS coupled with GNPS molecular networking to annotate molecular cluster nodes and preliminarily identified several known xanthone compounds. Notably, multiple unresolved nodes in the molecular network suggested the possible existence of xanthone hybrid metabolites containing unknown structural units. Targeted fractionation of xanthone-enriched components ultimately yielded 11 xanthone alkaloids, sydoxanthones F-M (**275–282**). Among these, compound **277a** exhibited significant neuroprotective activity by effectively scavenging H_2_O_2_-induced ROS and improving the viability of SH-SY5Y neuronal cells [112].

In addition, the genome mining techniques was used to systematically screen mangrove-derived fungi. Using the polyketide synthase gene AusA from *Aspergillus calidoustus* as a molecular probe, researchers identified a homologous gene cluster (tam) in the endophytic fungus *Talaromyces* sp. JNQQJ-4 isolated from *Kandelia candel*. GNPS molecular network analysis annotated two known DMTD analogs and several unresolved nodes. Through targeted isolation, seven structurally unique 3,5-dimethylorsellinic acid-derived meroterpenoids, talaromeroterpenoids A-G (**283‒289**) were obtained. Among them, compound **283** featured an unprecedented 7/6/6/6/5/5 hexacyclic system based on a 2,8,18,21-tetraoxa-hexacyclo [12.5.2.1^3,12^.0^1,16^.0^4,10^.0^16,22^] docosane core scaffold, which represented the first  novel  natural products. Bioactivity evaluation demonstrated that compound **284** significantly inhibited the expression of pro-inflammatory factors and exerted anti-inflammatory effects by modulating the NF-κB signaling pathway [113].

#### Coupling NMR metabolomics with genome mining

An innovative strategy combining metabolomics and genomics was used to systematically investigate  natural products of the marine-derived *Streptomyces* sp. S063. Through NMR-based metabolomic analysis, rare characteristic signals with negative chemical shifts (*δ*_H_ -0.34, corresponding to a methylene carbon at *δ*_C_ 21.3) were observed for the first time in the bacterial extracts. Meanwhile, the characteristic peaks for amino acid α-hydrogens (*δ*_H_ 4.0–6.0/*δ*_C_ 45.0–75.0) and *N*-methylation signals (*δ*_H_ 2.7–3.5/*δ*_C_ 25.0–40.0). These findings collectively suggested the presence of structurally novel *N*-methylated peptide metabolites. Further genome mining revealed a unique nonribosomal peptide synthetase gene cluster (Cluster 20), which not only contained key piperazic acid biosynthesis genes (lenE and lenF) but also exhibited distinctive structural features (including 13 NRPS genes, 10 adenylation domains, and 5 *N*-methyltransferase domains), with less than 48% homology to known peptide gene clusters. Based on these discoveries, researchers successfully isolated and identified two novel piperazic acid-containing cyclic decapeptides—lenziamides D1 (**290**) and B1 (**291**). Notably, both compounds demonstrated moderate growth inhibitory activity against multiple human cancer cell lines (including HEL, H1975, and H1299) as well as the paclitaxel-resistant strain A549-taxol, with IC_50_ values ranging from 8 to 24 μM. This study not only uncovered a structurally unique class of microbial secondary metabolites but also provided potential candidate molecules for the development of novel antitumor drugs [114] (Fig. 3 and Additional file 1: Fig. S1).

### Machine-learning-driven strategy

#### AI-facilitated enzyme tailoring platform

**Fig. 6:**
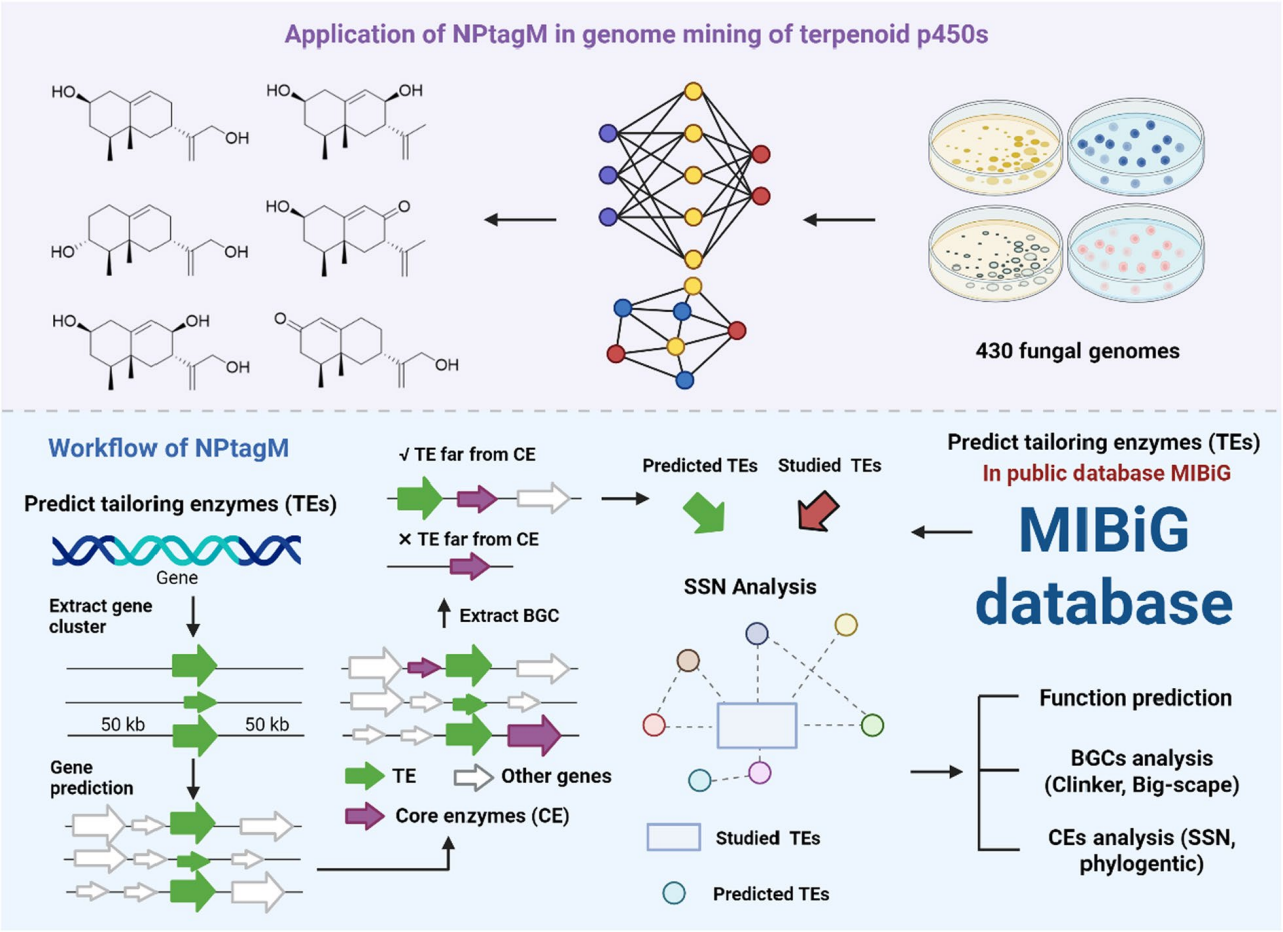
Framework of an AI-facilitated enzyme tailoring system

Machine-learning-driven identification of glycosyltransferase/P450 enzyme signatures for tailored natural product diversification. To overcome the limitations of traditional genome mining tools, the research team developed a novel genome mining toolkit, NPtagM, for modifying enzymes. By optimizing algorithmic architecture and workflows, this tool improved the prediction efficiency of P450 enzymes to three times that of the existing tool antiSMASH while reducing runtime by eightfold. Systematic analysis of fungal genomes using NPtagM identified a P450 enzyme family potentially involved in the biosynthesis of eremophilane-type sesquiterpenoids from 1,189 deduplicated terpenoid P450 enzymes. Heterologous expression in *Aspergillus oryzae* successfully yielded two novel eremophilane-type compounds (**292–293**) and four known analogues [115] (Fig. 6).

#### Intelligent CRISPR-guided genome engineering

Combinatorial deployment of Cas9 nickase with deaminases for DSB-free multiplexed engineering of biosynthetic machineries. Performance metrics in: in gene editing technology, the team developed a revolutionary multiplex base-editing (MBE) platform. By integrating the CRISPR-Cas9 system with base-editing tools, this platform achieved high-throughput gene editing in filamentous fungi, enabling simultaneous editing of up to eight genes in a single transformation. Using this technology, the team combinatorially regulated three epigenetic regulators (CclA, ClrD, and HdaA) in *Aspergillus nidulans*, successfully activating eight silent BGCs and isolating four structurally novel compounds, 8-oxycichorine (**294**), andicichorine A (**295**), andicichorine B (**296**), andicichorine C (**297**). In-depth studies revealed that the unique hybrid scaffolds of compounds **295**, **296**, and **297** likely originated from cross-reactions between the cichorine and polyamine metabolic pathways [116].

**Fig. 7:**
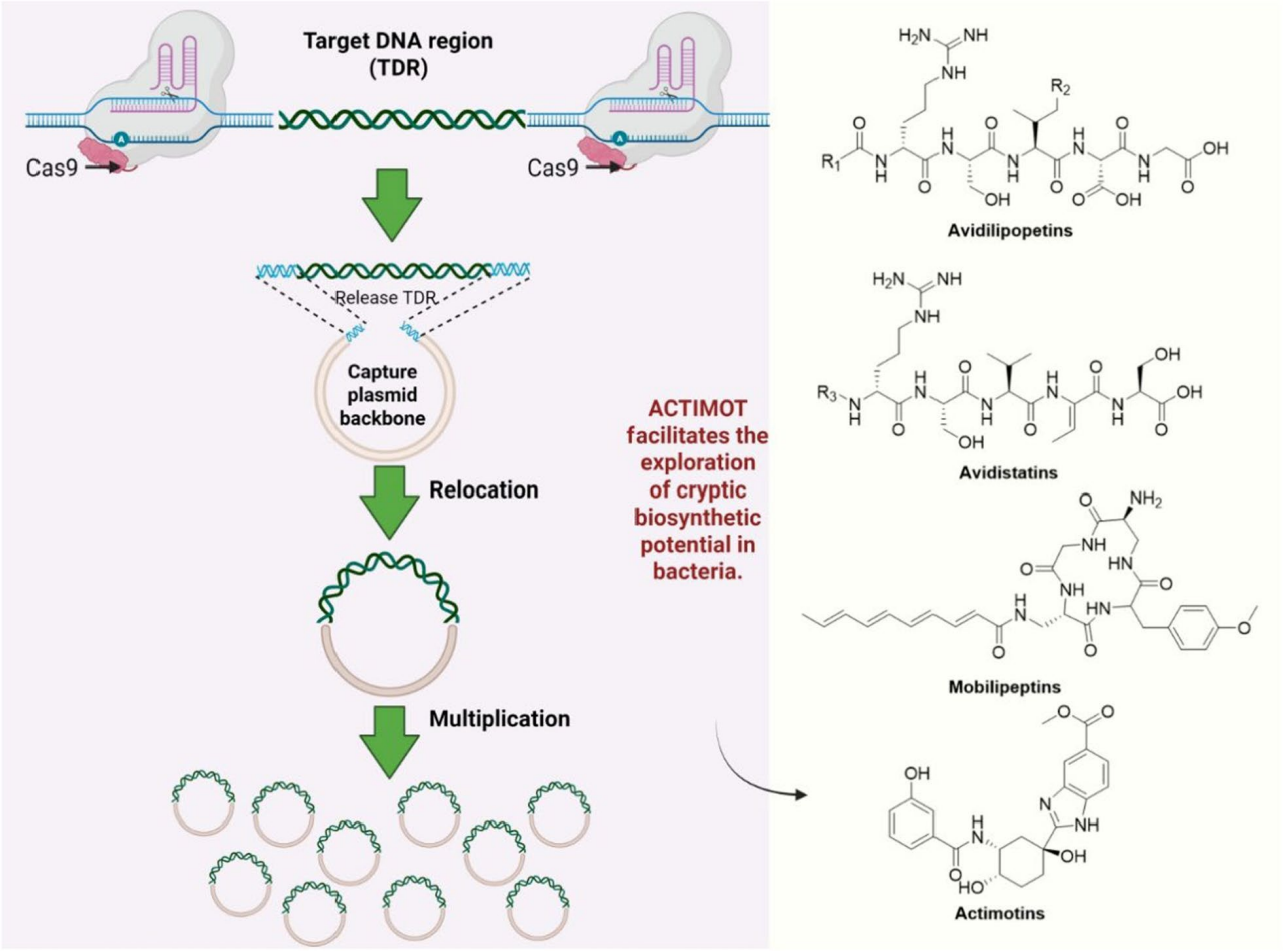
Framework of intelligent CRISPR-guided genome engineering

In addition, a CRISPR-Cas9-based gene editing tool named ACTIMOT was developed, which was inspired by the horizontal transfer mechanism of antibiotic resistance genes. This method enables the in situ excision and transfer of large chromosomal DNA fragments (such as silent biosynthetic gene clusters) directly within bacterial cells, efficiently integrating them into autonomously replicating plasmids. This circumvents the limitations of traditional cloning techniques in manipulating large DNA fragments. Applying this technology, the researchers successfully mobilized four cryptic gene clusters from the *Streptomyces* chromosome. Plasmid-based overexpression activated their biosynthetic pathways, ultimately leading to the discovery of 39 structurally diverse novel natural products (encompassing four distinct classes of compounds, avidistatins A1-A2, B1-B2, C1-C2, D (**298–304**), avidilipopeptides A1-A5, B1-B4, A6-A11 (**305–319**), mobilipeptins A-E (**320–324**), actimotins A-L (**325–336**)). his work demonstrates that ACTIMOT can accelerate the exploration of cryptic metabolic pathways and the discovery of natural products [117] (Fig. 7).

#### Structure-oriented intelligent screening

**Fig. 8:**
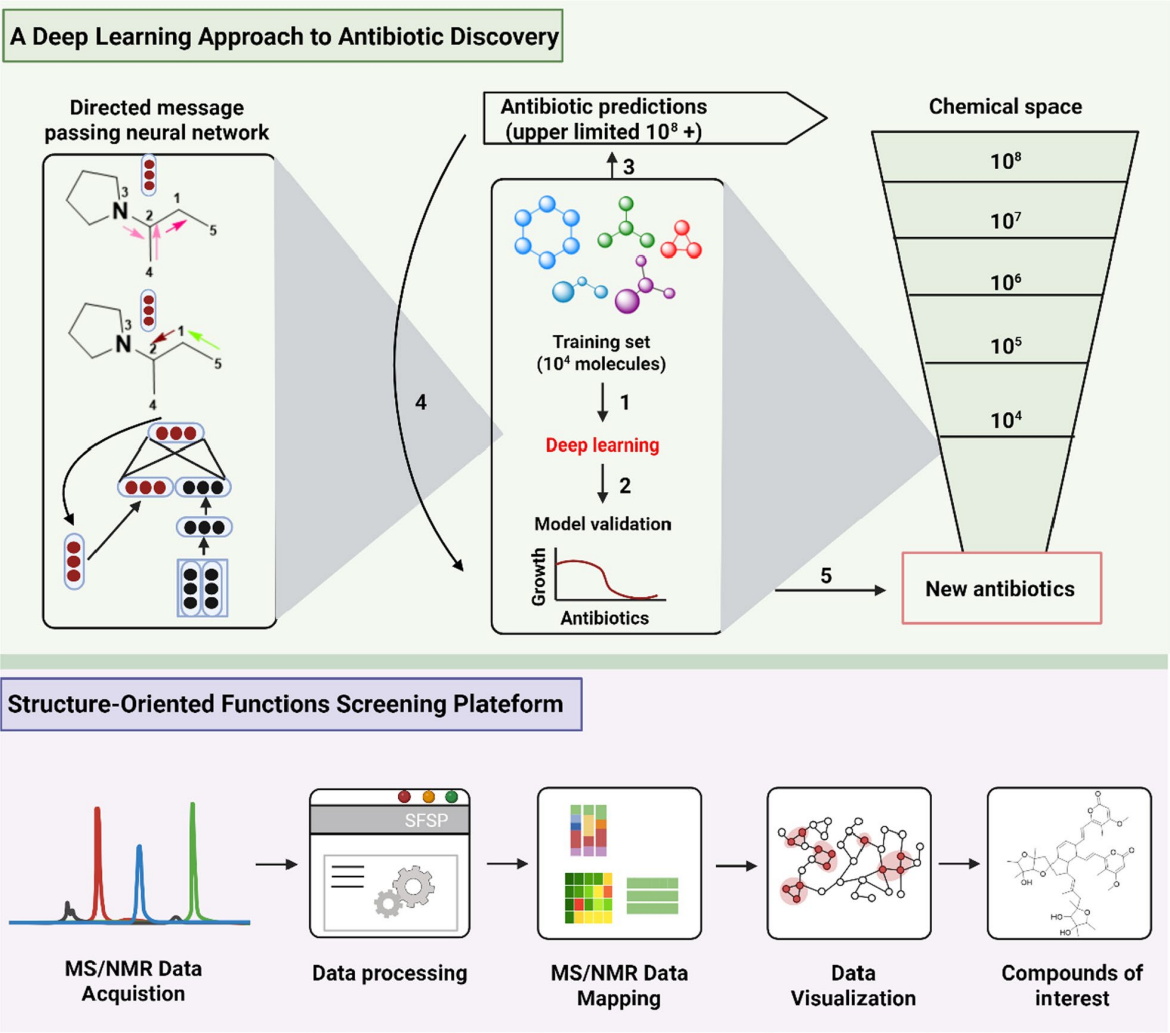
Workflow of structure-based intelligent screening

In this study, an antimicrobial activity prediction model was developed based on the deep neural networks. Initially, 2,335 known anti-*Escherichia coli* active molecules were trained. Then, the model was employed to direct message-passing neural networks to construct molecular representations and integrated multi-source features. After that, its performance was significantly enhanced due to the hyperparameter optimization and ensemble learning. The model was innovatively applied in a dual-track screening strategy: on one hand, it virtually screened over 107 million molecules from the ZINC15 database, leading to the experimental validation of 23 candidates and the identification of 8 novel antibacterial compounds after prediction score ranking; on the other hand, combined with targeted drug repositioning technology, it efficiently identified the structurally innovative antibiotic halicin (**337**) from the "Drug Repurposing Hub" library. Compound **337** possessing a markedly different structure from conventional antibiotics,  exhibited broad-spectrum of antimicrobial activity against clinically critical pathogens including *Mycobacterium tuberculosis* and carbapenem-resistant Enterobacteriaceae. Especially, compound 337 can be used to surpress *Clostridioides difficile* and treat pan-drug-resistant *Acinetobacter baumannii* infections in mouse models [118] (Fig. 8).

Structure-Oriented Fractions Screening Platform (SFSP) was established to be an efficient natural product discovery system by integrating machine learning algorithms with multidimensional spectroscopic data. Specifically, the SFSP platform enables direct prediction of compound node chemical shifts from extract library MS^1^ mass spectrometry data and ^1^H-PSYCHE-NMR spectra, achieving database-independent structural feature analysis. Using this innovative technological platform, we successfully identified two structurally novel classes of natural products from the deep-sea hydrothermal vent-derived fungus *Aspergillus* sp. GE2-6: Flavipidin-type compounds identified through ^1^H NMR olefin signal guidance, including flavipidins A-E (**338–342**), diflavipidins A-Q (**343–359**), and isoflavipidins A-B (**360–361**); Phenalenones-type compounds discovered via ^1^H NMR low-field hydroxy signal guidance: asperphenalenones N-P (**362–364**). Notably, compound** 338** demonstrated significant anti-influenza A virus (PR8 strain) activity (IC_50_: 21.9/12.9 μM), while compounds **363–364** effectively inhibited HIV pseudovirus infection in 293FT cells (IC_50_: 6.1/4.6 μM) [119] (Fig. 8).

## Summary and prospects

The development strategies for microbial natural products each have their own characteristics: the OSMAC strategy can activate silent gene clusters by simply adjusting culture conditions, but it suffers from uncontrollable products and low yields; the structure-based localization strategy is highly targeted but heavily relies on known compound structure databases, limiting its predictive capability for novel structures or rare modification patterns; the conservative mining strategy of core biosynthetic enzymes can rapidly locate key genes through conserved functional domains, making it suitable for high-throughput screening of homologous gene clusters, but it may miss atypical biosynthetic enzymes or functionally differentiated enzymes, and the conservative analysis cannot directly reflect the activity of gene clusters or the structural diversity of products, requiring experimental validation; in omics integration strategies, HPLC–MS offers high sensitivity but depends on existing mass spectrometry libraries, while NMR provides precise structural resolution but has limited sensitivity; in machine learning-driven intelligent strategies, the NPtagM targeted enzyme modification platform can automatically identify glycosylation, methylation, and other modifying enzymes, accelerating structural derivatization, but it relies on training sets of known modifying enzymes and performs poorly in predicting non-classical modifications; CRISPR base-editing technology can precisely activate silent gene clusters, directly establishing gene-product associations, making it suitable for hard-to-culture microorganisms; deep neural network prediction can learn complex structure–activity relationships, enabling virtual screening and significantly reducing experimental workload, but it requires large sample sizes for stable training.

The core contradiction in current research lies in the fact that while microbial genomic data is growing rapidly (with over 200,000 genomes sequenced), the proportion of systematic studies remains extremely low [120–122]. This gap highlights both the enormous potential and the significant challenges in the development of microbial secondary metabolites [123, 124]. Notably, microbial genetic diversity far exceeds expectations [125]. Taking fungi as an example, their genome size and number of gene clusters far surpass those of bacteria, but complex regulatory networks and bottlenecks in genetic manipulation severely hinder research progress [126].

Future breakthroughs should focus on three dimensions: at the technological innovation level, new cultivation techniques and single-cell analysis methods need to be developed to overcome the challenges of accessing unculturable microbial resources; in terms of strategy optimization, deeper integration of multi-omics is required, particularly the establishment of "genome-transcriptome-metabolome" correlation networks; the key to methodological innovation lies in interdisciplinary integration, including: (1) bioinformatics-driven intelligent prediction, (2) synthetic biology-guided rational design, and (3) microfluidic technology-enabled high-throughput screening. With breakthroughs in CRISPR and other technologies [27], research on microbial secondary metabolism has entered a golden development period. The construction of a "data-driven experimentation, experimental validation, and engineering optimization" research framework holds promise for delivering transformative breakthroughs in pharmaceutical development and agricultural applications.

## Supplementary Information

Additional file 1. Supplementary file contains structres of 364 compounds isolated from microbes through some innovative strategies

## Data Availability

No data was used for the research described in the article.
