# Supplementary material for: Revolutionizing microbial treasure troves: innovative strategies for natural products discovery
Source: Nat Prod Bioprospect. 2026 Jan 10;16(1):12. doi: 10.1007/s13659-025-00565-0 (PMC12790562; doi:10.1007/s13659-025-00565-0)
Supplement: Supplementary file 1 — Additional file 1. Supplementary file contains structres of 364 compounds isolated from microbes through some innovative strategies [file 13659_2025_565_MOESM1_ESM.docx]

**Supplementary Information**

**Revolutionizing microbial treasure troves: innovative strategies for natural products discovery**

Yu-Jie Li,^1,2,3^ Ming-Hua Qiu,^1,2^ Xing-Rong Peng^1,2*^

^1^ *State Key Laboratory of Phytochemistry and Natural Medicines, Kunming Institute of Botany, Chinese Academy of Sciences, Kunming 650201, China*

^2^ *Kunming College of Life Science, University of Chinese Academy of Sciences, Kunming 650204, China*

^3^ *Key Laboratory of State Forestry and Grassland Administration on Highly-Efficient Utilization of Forestry Biomass Resources in Southwest China, Southwest Forestry University, Kunming 650224, P. R. China*

^*^*Corresponding authors: Telephone: +86-871-65223327,* *Fax: +86-871-65223325, E-mail:* [*pengxingrong@mail.kib.ac.cn*](mailto:pengxingrong@mail.kib.ac.cn)

**Fig. S1** Structures of microbial natural products (reported 2019-2025).
